# GSK3β inhibition and canonical Wnt signaling in mice hearts after myocardial ischemic damage

**DOI:** 10.1371/journal.pone.0218098

**Published:** 2019-06-20

**Authors:** Lina Badimon, Laura Casaní, Sandra Camino-Lopez, Oriol Juan-Babot, Maria Borrell-Pages

**Affiliations:** 1 Cardiovascular Program ICCC, Institut de Recerca de l’-Hospital de la Santa Creu i Sant Pau, Barcelona, Spain; 2 CIBER-CV, Instituto de Salud Carlos III, Madrid, Spain; 3 Cardiovascular Research Chair, UAB, Barcelona, Spain; National Cancer Center, JAPAN

## Abstract

**Aims:**

Myocardial infarction induces myocardial injury and tissue damage. During myocardial infarction strong cellular response is initiated to salvage the damaged tissues. This response is associated with the induction of different signaling pathways. Of these, the canonical Wnt signaling is increasingly important for its prosurvival cellular role, making it a good candidate for the search of new molecular targets to develop therapies to prevent heart failure in infarcted patients.

**Methods:**

Herein we report that GSK3β regulates the canonical Wnt signaling in C57Bl6 mice hearts. GSK3β is a canonical Wnt pathway inhibitor. Using GSK3β inhibitors and inducing myocardial injury (MI) in *Lrp5*^*-/-*^ mice model we show that GSK3β phosphorylation levels regulate downstream canonical Wnt pathway genes in the ischemic heart. In the setting of MI, myocardial damage assessment usually correlates with functional and clinical outcomes. Therefore, we measured myocardial injury size in *Wt* and *Lrp5*^*-/-*^ mice in the presence and absence of two different GSK3 inhibitors prior to MI. Myocardial injury was independent of GSK3 inhibitor treatments and GSK3β expression levels.

**Results:**

These studies support a central role for GSK3β in the activation of the canonical Wnt pathway in the *Wt* heart. Although LRP5 is protective against myocardial injury, GSK3β expression levels do not regulate heart damage.

## Introduction

Acute myocardial infarction leads to acute cardiac ischemic injury. Myocardial infarction usually initiates with complete coronary artery occlusion because of the rupture of an atherosclerotic plaque and the subsequent thrombotic process [1]. The extent of the cardiac injury depends on the duration and location of the obstruction of the blood flow. Indeed, cardiomyocytes in the cardiac tissue downstream from the blocked vessels can be killed within minutes causing a massive and immediate inflammatory response that will clear the injured tissue. This response will promote the formation of a sparse cellular tissue that will be filled in by different cellular processes that include infiltration of myofibroblasts, which will deposit collagen and other extracellular matrix proteins, and activation and proliferation of endothelial cells [2]. In an attempt to restore blood supply, new vessels will be formed in a process named angiogenesis. Approximately a week after the initial ischemic assault a scar will form in the cardiac tissue. The low regenerative capacity of the heart coupled to the extensive systemic response to preserve ventricular integrity will eventually cause a permanent loss of cardiac tissue that will lead to ventricular remodeling and heart failure. Sometimes, efficient and synchronous heart contraction is compromised by excessive scar formation that acts as a barrier to allow a correct electromechanical signaling between the healthy regions of the heart. A better understanding of the cellular and molecular mechanisms involved in cardiac repair could help design strategies to delay the onset of heart failure in infarcted patients.

During embryonic development Wnt signaling, an intracellular pathway that regulates vertebrate cardiomyocyte differentiation[3], can act through three different mechanisms: a β-catenin dependent pathway called canonical Wnt pathway, and two β-catenin independent pathways called noncanonical Wnt pathways [3,4]. The canonical Wnt pathway is activated when an extracellular Wnt ligand binds to a seven-pass transmembrane Frizzled (Fz) receptor and its co-receptor low-density lipoprotein receptor related protein 5 (LRP5) or LRP6. Extracellular lipids have also been shown to activate canonical Wnt signaling [5]. Ligand binding to the Wnt receptors induces the recruitment of the cytoplasmic scaffolding protein Dishevelled (Dvl) resulting in LRP5 phosphorylation and the recruitment of the cytoplasmic axin complex to the plasma membrane. The axin complex is composed of the scaffolding protein Axin, the tumor suppressor protein Adenomatous Polyposis Coli (APC), casein kinase 1 (CK1), and glycogen synthase kinase 3β (GSK3β). After the destruction of the axin complex, β-catenin is stabilized and accumulates in the cytoplasm until it eventually translocates to the nucleus to form complexes with the DNA-bound T Cell Factor/Lymphoid Enhancer Factor (TCF/LEF) family of transcription factors activating Wnt target gene expression. In the absence of a Wnt ligand, cytoplasmic β-catenin is constantly degraded by the axin complex. Indeed, GSK3β sequentially phosphorylates the amino terminal region of β-catenin, resulting in β-catenin recognition by an E3 ubiquitin ligase subunit, β-Trcp, and subsequent β-catenin ubiquitination and proteasomal degradation [6]. This continual elimination of β-catenin prevents β-catenin from reaching the nucleus, and Wnt target genes are thereby repressed. Several known target genes regulated by the canonical Wnt signalling pathway are: cyclo-oxygenase-2[7], c-jun [8], vascular endothelial growth factor (VEGF) [9], matrix metalloproteinase 7 (MMP7) [10], osteopontin (OPN) [11] and bone morphogenetic protein 2 (BMP2) [12].

We have recently described the prosurvival function of the canonical Wnt pathway in vascular cells of atherosclerotic lesions [5, 13–15] and in myocardial injury (MI) [16]. Expression of several Wnt pathway proteins such as β-catenin and Dvl are increased in the damaged tissue after experimental myocardial infarction-induction [17–20]. However there is an ongoing controversy on the effects of Wnt signaling activation on cardiac recovery after myocardial damage. Indeed, the overexpression of two Wnt signaling antagonists, secreted Fz-related protein 1 (sFRP1) and sFRP2 was reported to reduce infarct size and improve cardiac function, suggesting that blocking Wnt signaling is cardioprotective in mice [17, 21, 22]. In apparent contradiction, sFRP2 deficient mice subjected to ischemic damage show smaller infarcts and improved cardiac function [20]. Furthermore, the transfer of β-catenin (with a constitutively active adenovirus) in a rat myocardial infarction model induced a reduction in infarct size [23]. We have previously evidenced that mice lacking Lrp5 (*Lrp5*^-/-^ mice) show more myocardial damage than *Wt* mice [16] suggesting that canonical Wnt signaling activation is cardioprotective. A possible explanation for these apparent contradictory results is that experiments were performed in different animal models with different myocardial injury-induction times and used different parameters to analyze Wnt pathway modulation. A major regulator for the canonical Wnt pathway is GSK3β. GSK3β is an evolutionary conserved, ubiquitous serine/threonine kinase that participates in several signaling pathways [24–26]. To date, numerous studies have indicated that GSK3β acts as a downstream regulator that determines the outcome of several prominent pathways following stimuli such as Wnt signaling, phosphatidylinositol 3-kinase, and neurotrophic pathways. The diverse functions of GSK3β include the regulation of circadian rhythms [27] and cell structure, and survival [26, 28]. Deregulation of GSK3β has been involved in diabetes, schizophrenia, Alzheimer disease and cancer [26, 29]. However, the precise signaling mechanisms in which GSK3β is involved are not well understood and it is an area of active research. GSK3β is unique because it is constitutively active in cells in resting conditions where it is primarily regulated by inhibition of its activity. GSK3β activity is positively regulated by phosphorylation of Tyr^216^ and negatively regulated by N-terminal phosphorylation of Ser^9^ [25]. Indeed, increased phophorylation in Tyr^216^ of GSK3β in neuronal cells correlated with an increase in GSK3β activity [30, 31]. Furthermore inhibition of GSK3β Tyr^216^ phosphorylation decreased the survival and/or proliferation of different types of cancer, both *in vitro* and *in vivo* [32, 33]. To further understand the role of the canonical Wnt pathway in myocardial injury we have investigated the role of GSK3β in the heart of *Lrp5*^*-/-*^ and wild type mice.

## Materials and methods

### Nomenclature

Mice genes are written in italics (*Lrp5*) and mice proteins in straight capital block letters (LRP5) in accordance with the guidelines from the “International Committee on Standardized Genetic Nomenclature for Mice and the Rat Genome”, 2010.

### Animals and experimental design

All procedures were approved by the Institutional Animal Care and Use Committee (CEEA-ICCC) and approved by local government animal experimentation with number 5422 on 10/05/2010. The study protocols for mice were approved by the institutional animal care and use committee (ICCC051⁄5422) and authorized by the local government commission. All animal procedures follow the guidelines from Directive 2010/63/EU of the European Parliament and the “Position of the American Heart Association on Research Animals use” (November 11, 1984). Therefore all animal studies have been approved by the ethics committee of our Hospital and have been performed in accordance with the ethical standards laid down in the 1964 Declaration of Helsinki and its later amendments. At the Hospital de la Santa Creu i Sant Pau we are committed to the “3Rs” principle and hence used the minimum of animals required to achieve statistical significance. *Lrp5*^*-/-*^ mice, a kind gift from Dr. Bart Williams [34–36], were maintained in a C57BL/6 background. Mice were housed in cages under controlled temperature (21±2°C) on a 12h light/ dark cycle with food and water ad libitum. Homozygous wild type C57BL/6 mice (*Wt*) and *Lrp5*^*-/-*^ C57BL/6 mice (*Lrp5*^*-/-*^*)* were used in this study. The presence of Lrp5 alleles was assessed by PCR amplification from DNA extracted from tail biopsies in wild type, heterozygous and homozygous littermates. Primers used were S17 (GGC TCG GAG GAC AGA CCT GAG), S23 (CTG TCA GTG CCT GTA TCT GTC C) and IRES31 (AGG GGC GGA ATT CGA TAG CT). *Lrp5*^*-/-*^ and *Wt* mice were fed a normal chow diet (NC, Tekland diet, Harland Labs) for 18 weeks when experiments were performed. At sacrifice, cardiac puncture was performed in mice under terminal anesthesia (1mg/kg Medetomidine and 75mg/kg Ketamine, ip).

### Mice acute myocardial ischemic damage

For experimental myocardial injury (MI)-induction male mice were analgesized (buprenorphine 0.1mg/kg, i.p.), anesthetized with a mixture of ketamine (75mg/kg, i.p.) and medetomidine (1mg/kg, i.p.), intubated and mechanically ventilated (100% O_2_, 90 breaths/min; SAR-830; CWE Inc.). The heart was exposed through a medial incision of the chest and the left anterior descending (LAD) coronary artery was permanently occluded at the site of its emergence from under the left atrium with an intramural stitch (7–0 silk suture) for 60 minutes. Ischemia was confirmed by immediate bleach of the myocardium and momentary elevation of the ST wave in the ECG. Heart rate, ECG and body temperature were recorded throughout the experimental procedure using a Biosonic pad (Bioscan). At the end of the MI induction hearts were extracted, immersed in fixative solution (4% paraformaldehyde), embedded in OCT, cut into 5 μm thick serial sections and placed on poly-L-lysine coated slides. Hematoxylin and eosin and Masson's Trichrome staining was performed to visualize tissue structure and calculate the damaged area in the left ventricle and interventricular septum. Images were captured by Leica MZ95 stereo-microscope and digitized by Retiga 1300i Fast camera. A section every five cuts was analyzed and myocardial injury was measured by Image J software as the percentage of the damaged area in relation to the total left ventricle. In order to reduce variability, myocardial injury assessments were all performed by the same investigator and blindly to mice genotype.

One group of *Wt* and *Lrp5*^*-/-*^ mice was treated with CHIR99021 (15mg/kg, i.p.; Selleckchem; 90 minutes half life) a highly selective inhibitor of GSK3 1 hour prior to MI induction. Another group of *Wt* mice was treated with SB415286 (10mg/kg i.p; Sigma Aldrich; 20 hours half life), another GSK3 inhibitor with a longer half life than CHIR99021, 24h and 2h prior MI induction. After 60 minutes of MI-induction mice were euthanized, hearts were separated into ischemic and non-ischemic regions and frozen in liquid nitrogen. Another set of hearts were fixed in PFA, stained with haematoxylin-eosin and Masson Trichromic and myocardial damage analyses were performed. Sham-operated mice, which underwent the same surgical procedure without arterial ligation, served as controls. The organs were harvested for further analysis. Additionally, after MI induction, two groups: *Wt* and *Wt*+SB415286 mice underwent 90 minutes of reperfusion when the hearts were extracted, fixed in PFA or frozen in liquid nitrogen. A minimum of 6 mice/group was performed.

The study groups are summarized below:

*Wt* and *Lrp5*^*-/-*^ mice: 1h MI.
*Wt* and *Lrp5*^*-/-*^ mice: 1h CHIR99021+ 1h MI.
*Wt* mice: 24h SB415286 (two injections at 24h and 2 h prior MI) + 1h MI.
*Wt* mice: 1h MI+ 90 min reperfusion.
*Wt* mice: 24h SB415286 (two injections at 24h and 2 h prior MI) + 1h MI+ 90 min reperfusion.

### Real time RT-PCR

Ischemic and non ischemic regions of hearts of the different mice study groups were frozen in liquid nitrogen for gene and protein analyses. Myocardial RNA was isolated with Trizol Reagent (Invitrogen) (n = 5–7 mice/group). Concentration was determined with a NanoDrop ND-1000 spectrophotometer (NanoDrop Technologies, Inc., Wilmington, DE, USA) and purity was checked by the A260/A280 ratio (ratios between 1.8 and 2.1 were considered acceptable). cDNA was synthesized from 1 μg RNA with cDNA Reverse transcription kit (Qiagen). The resulting cDNA samples were amplified with a RT-PCR thermal cycler (Applied Biosystems 7900HT) and the following specific probes from Applied Biosystems: *Lrp5* (Mm01227476_m1), *Lef1* (Mm00550265_m1), *β-catenin* (Mm00483039_m1), *Vegf* (Mm00437306_m1) and *Gsk3β* (Mm00444911_m1). Results were normalized with 18S probe from Applied Biosystems.

### Western blot

Sample extracts (30μg protein) were resolved by SDS-PAGE and transferred to nitrocellulose membranes, blocked with 5% skim milk and probed for monoclonal GSK3β and LRP5 (Abcam), LEF1 (Millipore)or polyclonal pY216 GSK3β, VEGF (Abcam) primary antibodies at a concentration of 1μg/ml. Membranes were then washed and blotted with the appropriate secondary antibody (Dako). Band densities were determined with the ChemiDoc XRS system (Bio-Rad) in chemiluminescence detection modus and Quantity-One software (Bio-Rad). Normalization was performed against β-ACTIN.

### Statistical analysis

The Shapiro-Wilk test was applied to verify the normal distribution of data. Intergroup comparisons were performed by using unpaired Student *t* tests or analysis of variance tests followed by Fisher protected least significant difference as post hoc analysis. A p value of <0.05 was considered significant, and results are reported as mean ± SEM. All the experiments were performed at least three times. All statistical analyses were conducted using Statview and R version 3.3.0 software (R Foundation for Statistical Computing).

## Results

### p-GSK3β levels in hearts of *Wt* and *Lrp5*^*-/-*^ mice

To evaluate the response of Wnt pathway components to myocardial ischemic injury, we permanently occluded the left anterior descending (LAD) coronary artery of male C57BL/6J Wild Type (*Wt*) and male *Lrp5*^*-/-*^ mice for 1hour and sacrificed the animals. Another set of *Wt* and *Lrp5*^*-/-*^ male mice were administered CHIR99021 (15mg/kg, i.p.), 1 hour prior to LAD occlusion and sacrificed. CHIR99021 is a selective small molecule GSK3 inhibitor that activates Wnt signaling.

**Fig 1:**
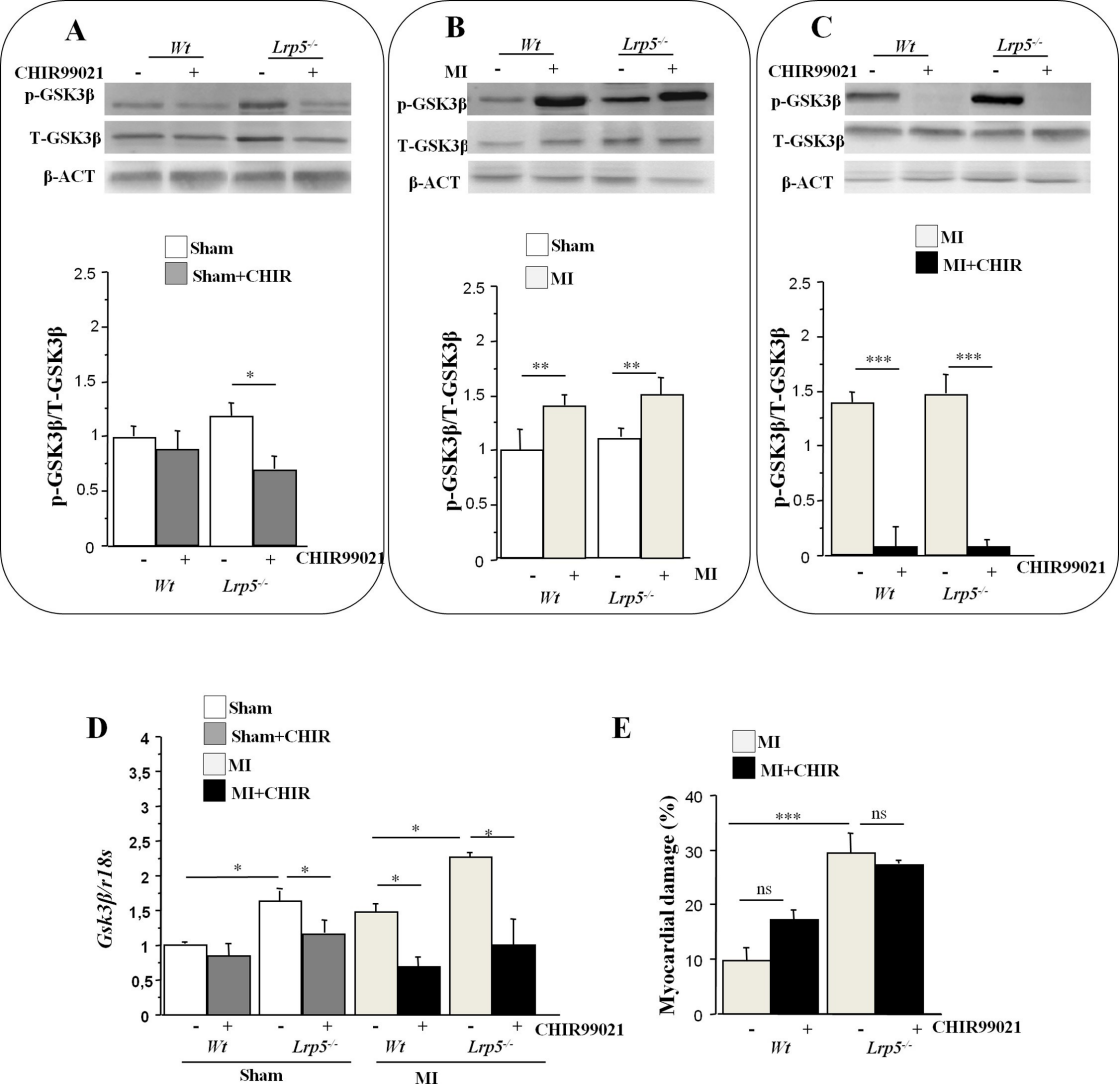
p-GSK3β levels in hearts of *Wt* and *Lrp5*^*-/-*^ mice. Representative WB of phosphorylated GSK3β at Tyr216, total GSK3β protein and β-ACTIN expression levels in ischemic hearts of *Wt* and *Lrp5*^*-/-*^ mice (A) with or without 15 mg/kg i.p. CHIR990021 treatments (B) after myocardial injury (MI)-induction and (C) after CHIR99021 treatments and MI induction. Bar graphs showing the quantification of the WB bands intensity p-GSK3β/T- GSK3β protein. (D) GSK3β mRNA expression levels in hearts of *Wt* and *Lrp5*^*-/-*^ mice with or without CHIR990021 treatments and MI induction. (E) Myocardial damage measurements of *Wt* and *Lrp5*^*-/-*^ mice after MI in the presence and absence of CHIR99021. 6–10 male mice /condition *p<0,05; ***p<0,005, ns: non significant.

Phosphorylated GSK3β (p-GSK3β, Tyr^216^) protein levels were higher in ischemic regions of hearts from *Lrp5*^*-/-*^ mice than in those from *Wt* mice (Fig 1A). Total GSK3β protein levels were also increased in the myocardium of *Lrp5*^*-/-*^ mice compared to *Wt* mice. We performed phosphorylated GSK3β/Total- GSK3β ratios to analyze total myocardial content of activated GSK3β and found no differences between genotypes (Fig 1A). CHIR99021 treatment significantly reduced p-GSK3β protein levels in *Lrp5*^*-/-*^ mice but not in *Wt* mice. CHIR99021 treatment reduced p-GSK3β to the same level in both genotypes. Induction of myocardial injury (MI) causes a highly significant increase of p-GSK3β protein levels in *Wt* and *Lrp5*^*-/-*^ mice, strongly suggesting that injury affects the canonical Wnt pathway (Fig 1B). Treatment with CHIR99021 before occlusion significantly reduced p-GSK3β levels in both genotypes (Fig 1C) further demonstrating efficacy of CHIR99021 treatments. Indeed, levels of p-GSK3β protein were significantly reduced after CHIR99021 treatments in damaged hearts leaving the canonical pathway open for activation and for β-catenin interaction with specific transcription factors.

We then assessed the expression GSK3β using real-time quantitative reverse transcriptase (RT)-PCR analysis. Results followed a similar pattern as protein expression indicating that CHIR99021 treatments are effective in inhibiting GSK3β myocardial gene expression in both mice genotypes (Fig 1D).

### Myocardial injury in *Wt* and *Lrp5*^*-/-*^ mice after CHIR99021 treatments

Myocardial damage, measured blindly as previously done in [16], was significantly larger in *Lrp5*^*-/-*^ mice than in *Wt* mice. However myocardial damage assessment was independent of CHIR99021 treatments and GSK3β levels. Indeed, reduction of GSK3β protein or gene levels did not have any effect on myocardial injury size.

### Wnt signaling in hearts of mice

**Fig 2:**
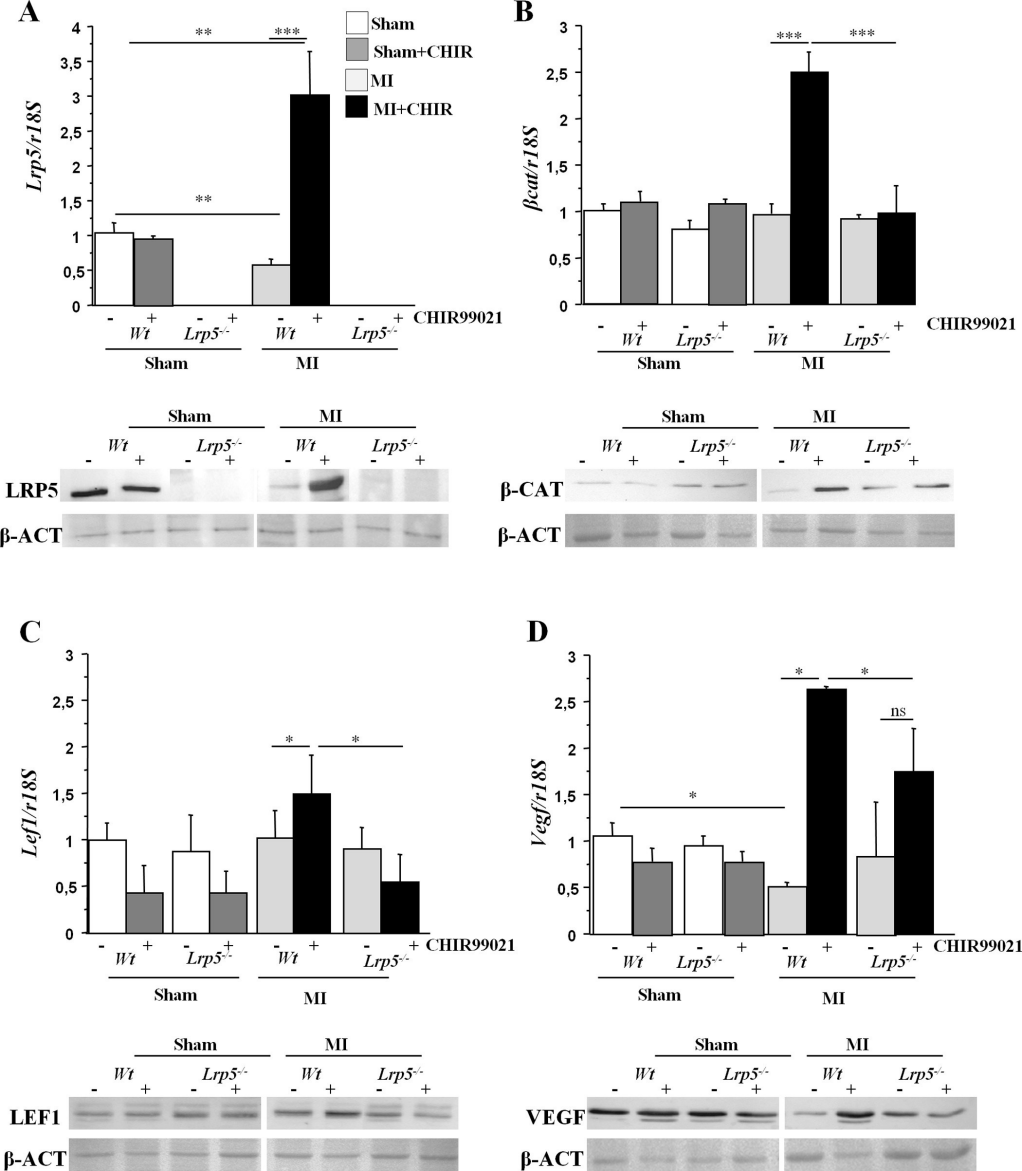
Canonical Wnt signaling proteins expression in hearts of *Wt* and *Lrp5*^*-/-*^ mice. mRNA expression levels and representative images of western blots showing protein expression levels of (A) *Lrp5*, (B) *β-catenin*, (C) *Lef1* and (D) *Vegf* in hearts of *Wt* and *Lrp5*^*-/-*^ mice. 6–10 male mice /condition *p<0,05; **p<0,01; ***p<0,005; ns: non significant.

We then analyzed components and targets of the canonical Wnt signaling pathway in the ischemic myocardium of *Wt* and *Lrp5*^*-/-*^ mice. In *Wt* mice, *Lrp5* mRNA and protein expression levels were downregulated immediately after MI-induction compared to sham animals (Fig 2A). However, expression of genes and proteins downstream GSK3β including *β-catenin*, *Lef1* and *Vegf* maintained similar expression in sham and MI induced *Wt* and *Lrp5*^*-/-*^ mice (Fig 2B–2D). After CHIR99021 treatments *βcatenin*, *Lef1* and *Vegf* gene and protein levels were increased in MI *Wt* mice (Fig 2B–2D). Interestingly, CHIR99021 treatments induced an increase in *Lrp5* mRNA levels after MI although its target is downstream the receptor. Similarly to *Wt* mice, in *Lrp5*^*-/-*^ mice, downstream Wnt signaling proteins were not induced in sham or MI-mice (Fig 2B–2D). There were no significant differences in gene expression levels between the myocardium of sham operated mice and non ischemic regions of MI-induced mice (S1 Fig).

### Myocardial damage in *Wt* mice after ischemia reperfusion

We then performed experiments aimed to determine the contribution of myocardial reperfusion to the modulation of the Wnt pathway. To this end we induced 60 minutes MI to *Wt* mice followed by 90 minutes reperfusion when animals were sacrificed (MI+Rep). Again, *Wt* mice which underwent the same surgical procedure without arterial ligation, served as controls (sham). A set of animals were treated 24h and 2h prior to MI induction with SB415286, a potent, selective and cell permeable GSK3 inhibitor with approximately 20 hours of half life ([37] CHIR99021 has a half life of approximately 90 min).

**Fig 3:**
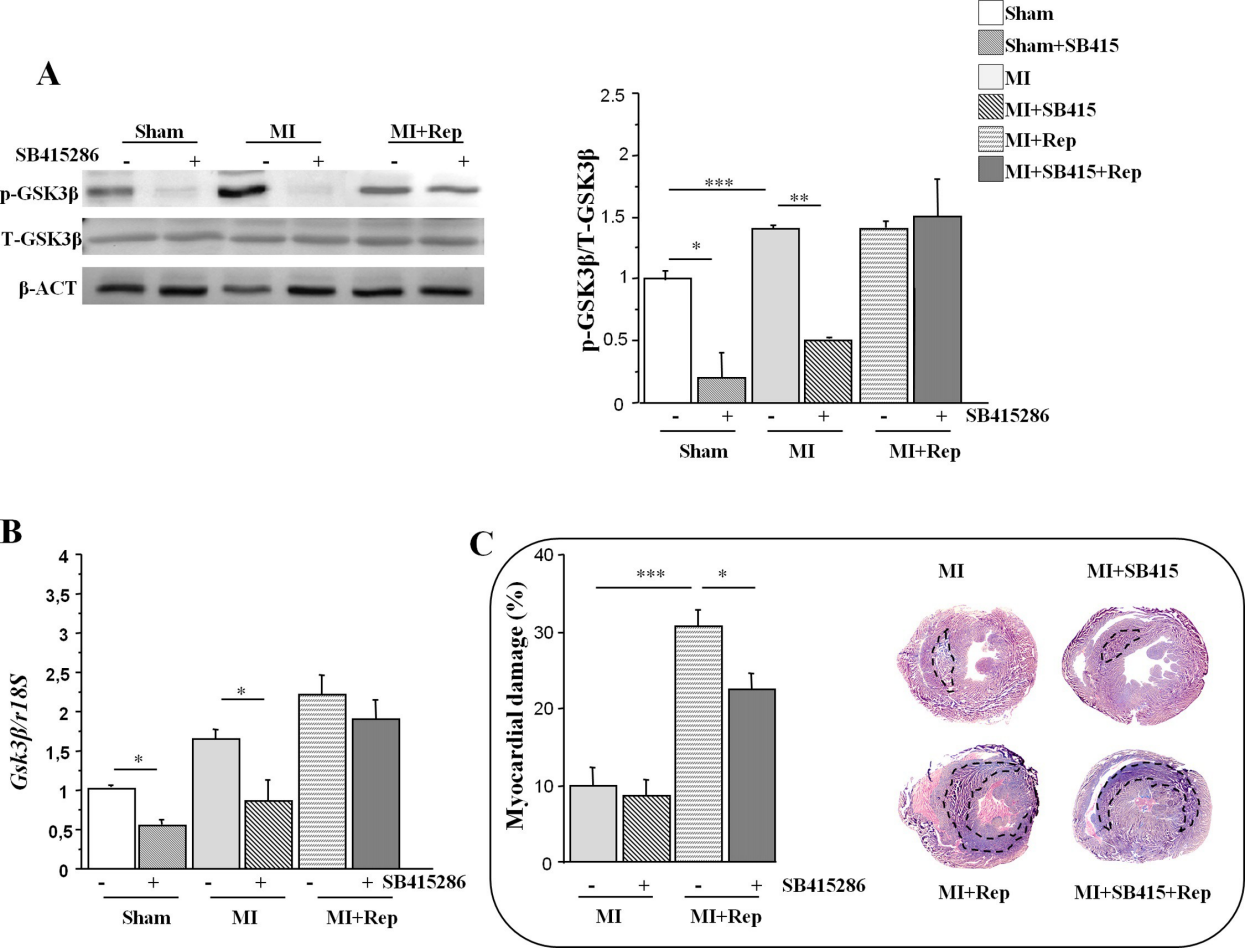
p-GSK3β in ischemic hearts of *Wt* and *Lrp5*^*-/-*^ mice. (A) Protein levels of phosphorylated and total GSK3β in hearts of *Wt* mice with or without 10mg/kg i.p. SB415286. p-GSK3β/T-GSK3β ratio of WB band intensities normalized with β-ACTIN. (B) mRNA expression (C) Myocardial damage assessment and representative images of *Wt* mice after MI and MI+Rep in the presence and absence of SB415286. 6–10 mice /condition *p<0,05; **p<0,01; ***p<0,005.

Results showed decreased p-GSK3β protein levels after SB415286 treatments in sham and MI-induced *Wt* animals. p-GSK3β (Tyr^216^)/T-GSK3β ratios are also shown (Fig 3A). GSK3β mRNA expression levels in *Wt* mice previously treated with SB415286 were also reduced as compared with untreated animals (Sham: 60.8% and MI: 51%; Fig 3B). However no reduction in p-GSK3β expression levels could be observed in the myocardium of reperfused mice (Fig 3A).

Measurements of myocardial injury show a 20% increase in *Wt* mice MI+Rep as compared to mice without reperfusion (Fig 3C). SB415286 treatments only reduced myocardial damage by 8% after reperfusion (Fig 3C).

### Wnt genes expression in hearts of *Wt* mice after reperfusion

**Fig 4:**
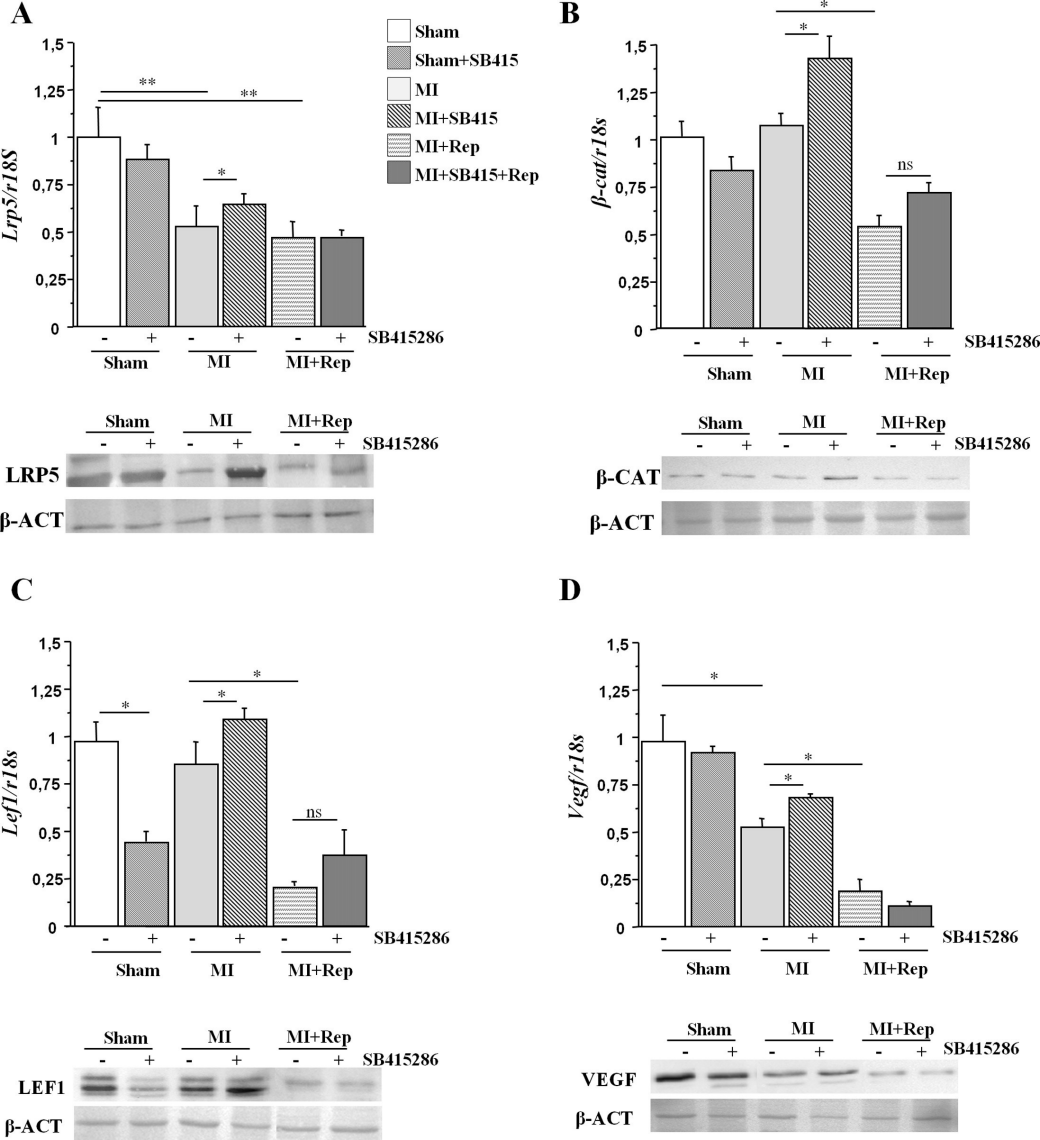
Canonical Wnt signaling protein expression in hearts of *Wt* mice after reperfusion. mRNA expression levels and representative images of protein expression levels of (A) *Lrp5*, (B) *β-catenin*, (C) *Lef1* and (D) *Vegf* in hearts of *Wt* mice after 90 minutes reperfusion in the presence or absence of SB415286. 6–10 mice /condition *p<0,05; **p<0,01; ***p<0,005; ns = non significant.

Analyses of Wnt signaling genes after MI in hearts of *Wt* mice show decreased *Lrp5*and *Vegf* gene and protein expression after MI-induction compared with sham operated animals (Fig 4A and 4D). *Wt* mice with ischemia-reperfusion treatments showed reduced canonical Wnt gene expression levels compared to non-reperfused MI-mice in *Lrp5* (19%), *β-catenin* (51%), *Lef1* (70%) and *Vegf* (60%; Fig 4A–4D). SB415286 treatments prior to MI in *Wt* mice increased the expression levels of the canonical Wnt pathway genes and proteins tested (Fig 4A–4D). However, SB415286 treatments in reperfused mice did not induce modifications in the expression levels of *Lrp5*, *β-catenin*, *Lef1* or *Vegf* as compared to MI+Rep mice.

## Discussion

We have extensively studied the role of LRP5 and the canonical Wnt pathway in *Lrp5*^*-/-*^ mice. We have shown that LRP5 is involved in several cellular processes including inflammation, monocyte differentiation, dislypidemia and atherosclerosis progression [5, 14, 15, 38]. Activation of canonical Wnt signaling pathway by extracellular Wnt ligands or lipids is inhibited in aortas of *Lrp5*^*-/-*^ mice but not in *Wt* mice [13]. Similarly, activation of the Wnt pathway is also abolished in circulating white cells of *Lrp5*^*-/-*^ mice [14]. Here we show that *Lrp5*^*-/-*^ mice have increased myocardial GSK3β transcription and protein levels opening the possibility that the previously observed inhibition of the canonical Wnt pathway in aortas and circulating white cells of *Lrp5*^*-/-*^ mice may be due to synergic effects of the lack of LRP5 and the increased GSK3β levels. However, further work is needed to confirm this hypothesis.

**Fig 5:**
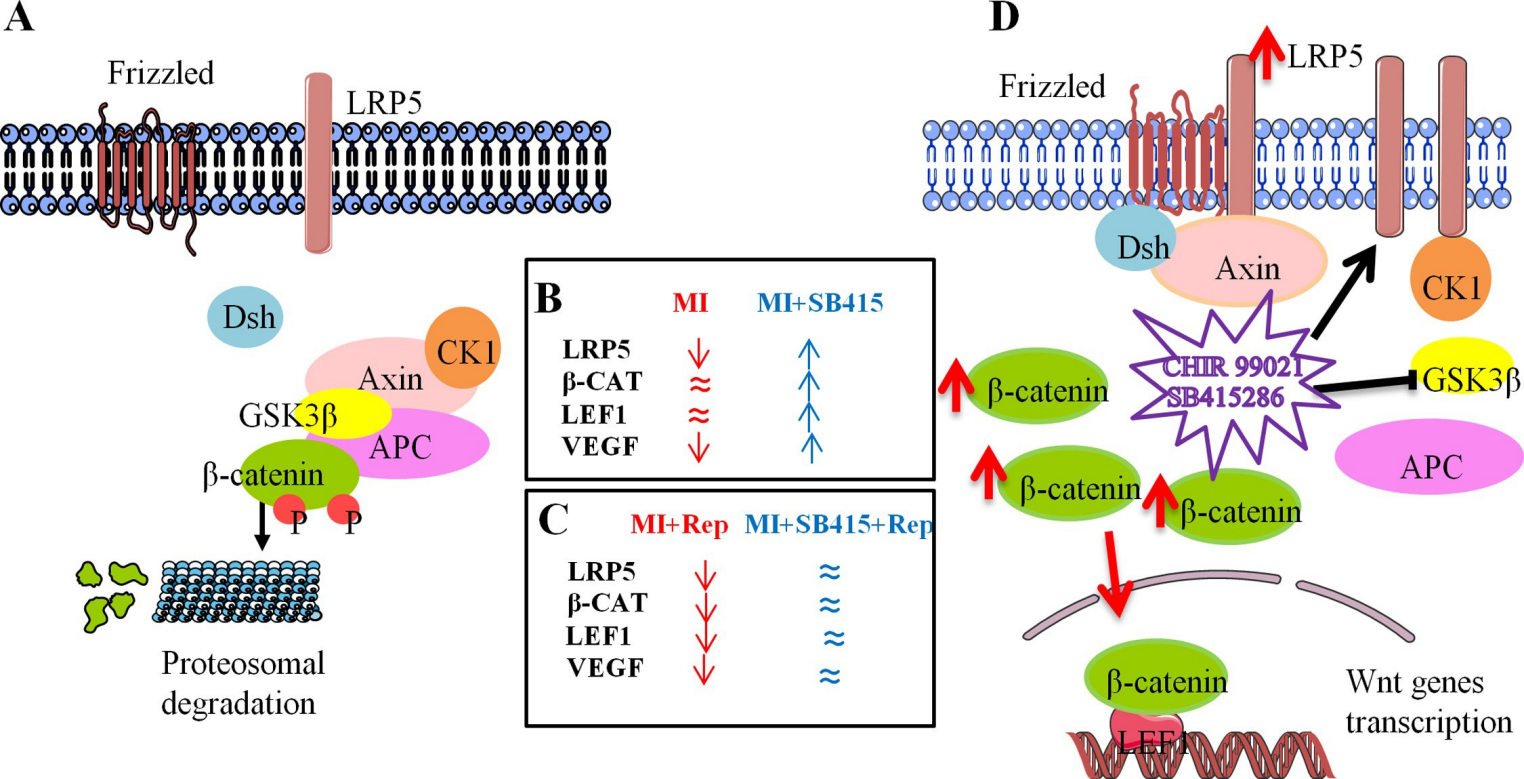
Schematic drawing of canonical Wnt signaling and GSK3β inhibitors. (A) Inactive canonical Wnt signaling: β-CATENIN is recruited into a cytoplasmic complex with AXIN, CASEIN KINASE 1 (CK1), ADENOMATOUS POLIPOSIS COLI (APC) and GSK3β that phosphorilates β-CATENIN targeting it for proteasomal degradation. (B) Protein levels of canonical Wnt proteins in hearts of *Wt* mice Sham vs MI or MI vs MI+SB415. (C) Protein expression levels of canonical Wnt proteins in hearts of *Wt* mice Sham vs MI+Rep or MI+Rep vs MI+SB415+Rep. (D) GSK3β inhibitors (CHIR99021 and SB415286) induce canonical Wnt signaling activation. β-CATENIN accumulates in the cytoplasm, translocates to the nucleus, binds to lymphocyte enhancer binding factor 1 (LEF1) family and initiates Wnt genes transcription.

Mice results show that phosphorylated GSK3β myocardial levels regulate Wnt pathway activation in *Wt* mice. Indeed, MI-induced *Wt* and *Lrp5*^*-/-*^ mice show high p-GSK3β levels without upregulation of canonical Wnt genes. The administration in *Wt* mice of CHIR99021, a canonical Wnt pathway activator, downregulated myocardial p-GSK3β levels and the expression of canonical Wnt genes increased (Fig 5). Several canonical Wnt pathway proteins and targets have been previously shown to be modulated in short periods of time. Indeed, β-catenin was increased as soon as 30 minutes after estradiol treatment in neuroblastoma cell lines [39], and VEGF protein expression in rat muscular fibres was significantly increased after only 90 minutes of exercise [40]. Other proteins that activate other signalling pathways are also regulated in short periods of time. Indeed, induction of c-*fos* mRNA was observed in mice ischemic cortex after 60 minutes of ischemia [41], and JAK 3 protein expression was significantly increased following 30 minutes of IL-2 stimulation and was sustained after 60 minutes in murine podocytes [42].

Long term GSK3β inhibition, induced by SB415286 administration downregulated p-GSK3β expression in the heart of *Wt* sham and MI-induced mice. Similar to CHIR99021 administration in *Wt* mice, SB415286 treatments induced an increase in Wnt signaling gene expression. Indeed, sham operated mice did not show Wnt gene expression variation before and after CHIR99021 or SB415286 treatments suggesting that the Wnt pathway is maintained in an inactive state until an injury occurs. This is sustained by our previous results where human macrophages in control conditions show an inactive Wnt signaling [5]. However, after LDL treatments, and upon Wnt pathway activation, β-CATENIN is translocated to the nucleus where it initiates canonical Wnt signaling genes transcription [5]. Unexpectedly, SB415286-treated *Wt* mice that had reperfusion after MI did not show reduced myocardial p-GSK3β levels. Accordingly Wnt signaling genes variations are minimal. These results further support that p-GSK3β levels regulate myocardial Wnt signaling in *Wt* mice and suggest that reperfusion inhibits GSK3β protein downregulation.

Previous studies on GSK3 inhibition on ischemia/reperfusion injury in *Wt* animals reported protective effects of GSK3 inhibitors in rabbits and rat hearts, in isolated perfused hearts and in isolated cardiomyocytes [43–47]. However, GSK3 inhibitors have also been reported not to induce protection in isolated perfused C57BL/6 mouse hearts using a global model of ischemia in a Langendorff heart [48]. Our results support a reduction in myocardial damage after the administration of GSK3 inhibitors in *Wt* ischemia reperfused mice. However, this does not correlate with reduced GSK3β protein levels nor with the triggering of canonical Wnt activation.

Currently there is an unfinished debate on the effects of the activation of the canonical Wnt signaling in the heart after myocardial damage [16, 17, 20, 23]. Indeed, transgenic mice that express a constitutively active form of GSK3β show diminished hypertrophy of cardiomyocytes in response to pressure overload [49]. Also, inhibition of GSK3β in perfused rat hearts after ischemic preconditioning resulted in the accumulation of β-catenin in the cytosol and nucleus [50]. Furthermore overexpression of sFRP1, an antagonist of the Wnt pathway, in mice with ischemic preconditioning after MI induced the activation of GSK3β [18]. However, permanent coronary artery ligation with no reperfusion in mice that overexpress sFRP1 showed reduced GSK3β activity [17]. Therefore, although the modulation of upstream signals of GSK3β in myocardial-ischemia has been investigated previously, myocardial ischemia models were different, and none of these studies inhibited LRP5 directly. In our *in vivo* non reperfused mice model we treated mice with an activator of the canonical Wnt pathway for 24h (long term) or 1h (short term) prior to MI induction and observed an increase in Wnt signaling gene expression. However, after 90 minutes reperfusion there was no reduction in the phosphorylation status of GSK3β and canonical Wnt signaling genes variations were minimal suggesting that reperfusion inhibits GSK3β protein downregulation. Our results show that GSK3β expression does not correlate with myocardial injury size in *Wt* mice. Indeed a reduction in p-GSK3β expression levels in mice that followed CHIR99021 or SB415286 treatments prior to MI did not induce reduced myocardial damage. Furthermore, *Wt* mice treated with CHIR99021 prior to MI had increased myocardial damage than those untreated. Similarly, *Lrp5*^*-/-*^ mice after CHIR99021 treatments did not show reduced myocardial injury size.

Therefore, we conclude that GSK3β plays a central role in the activation of the canonical Wnt pathway in the heart in *Wt* mice. However, GSK3β expression levels do not predict the outcome of myocardial damage.

## Supporting information

S1 Fig Canonical Wnt signaling genes expression in ischemic and non ischemic regions of *Wt* and *Lrp5*^*-/-*^ mice. mRNA expression levels of (A) *Lrp5*, (B) *β-catenin*, (C) *Lef1* and (D) *Vegf* in ischemic (I) and non ischemic (NI) regions of myocardium of *Wt* and *Lrp5*^*-/-*^ mice. 6–10 mice /condition *p<0,05; **p<0,01; ***p<0,005; ns: non significant.
(TIF)
