# Supplementary figures and images for: GSK3β inhibition and canonical Wnt signaling in mice hearts after myocardial ischemic damage
Source: PLoS One. 2019 Jun 20;14(6):e0218098. doi: 10.1371/journal.pone.0218098 (PMC6586285; doi:10.1371/journal.pone.0218098)

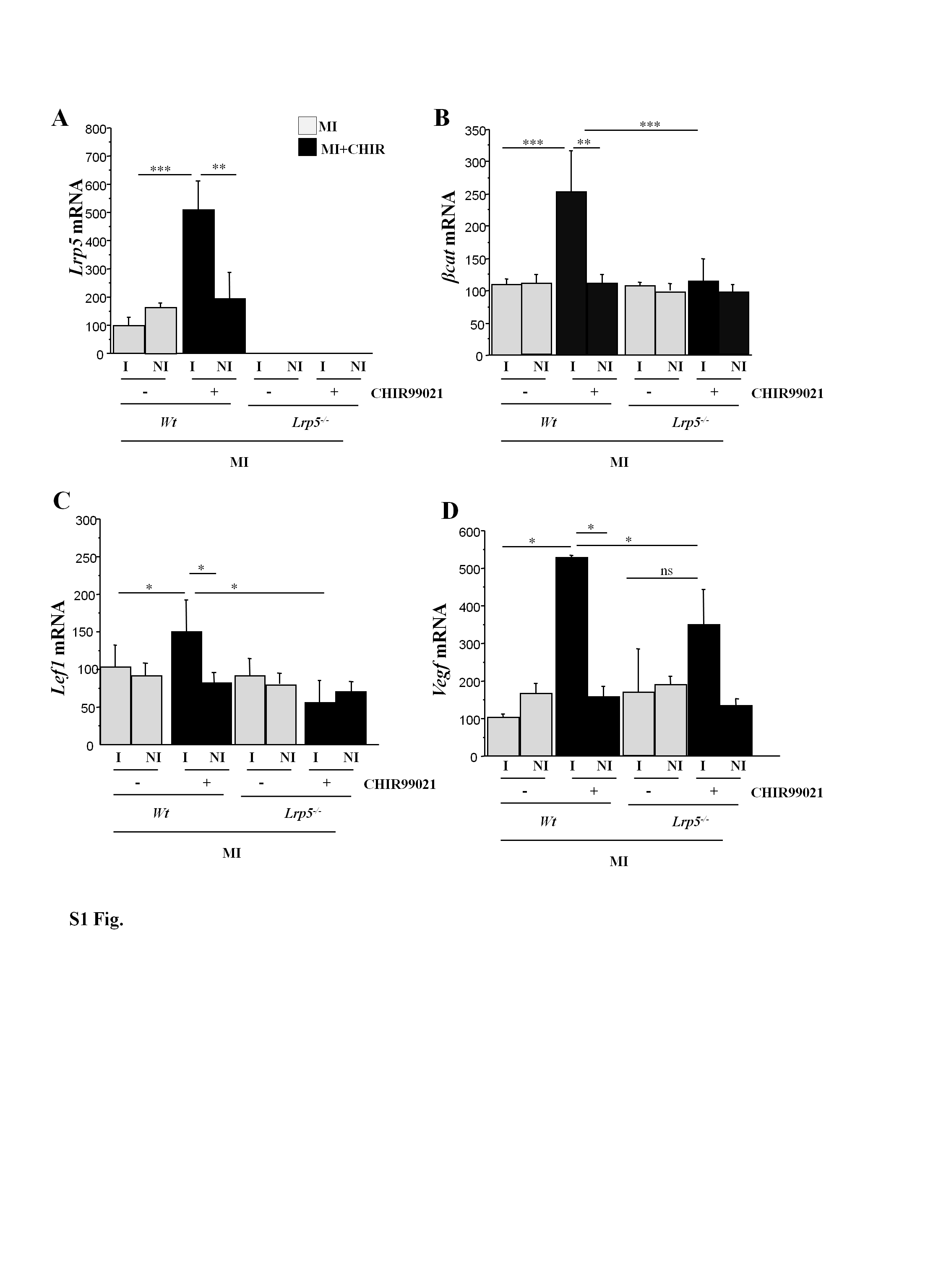

Supplement: S1 Fig — mRNA expression levels of (A) Lrp5, (B) β-catenin, (C) Lef1 and (D) Vegf in ischemic (I) and non ischemic (NI) regions of myocardium of Wt and Lrp5-/- mice. 6–10 mice /condition *p<0,05; **p<0,01; ***p<0,005; ns: non significant. (TIF) [file pone.0218098.s001.tif]
